# MKP1: Jekyll and Hyde for E1A

**DOI:** 10.18632/aging.100918

**Published:** 2016-02-29

**Authors:** Francisco J. Cimas, Juan Luis Callejas-Valera, Ricardo Sanchez-Prieto

**Affiliations:** Unidad de Medicina Molecular, Laboratorio de Oncología Centro Regional de Investigaciones Biomédicas, Universidad de Castilla-La Mancha, 02006, Albacete, Spain

**Keywords:** MKP1, E1A, MAPK, cisplatin, chemotherapy

MAP Kinase Phosphatase 1 (MKP1) - also known as Dual Specificity Phosphatase 1 (DUSP1) or CL100 - is a known member of Serine/Threonine inducible nuclear phosphatase family. Interestingly, this subset of phosphatases acts as MAPKs counterpart allowing switching off MAPKs as p38MAPK, ERK1/2, ERK5 or JNK. It is well-known that MKP1 plays critical roles in different key biological processes ranging from the immune system up to cell differentiation. On the other hand, it is also known that MKP1 levels are altered in several tumours, such as bladder, ovarian, prostate, breast, colon, and non-small cell lung cancers (NSCLC) among others (for a review of MKP1 and cancer see [1]). For example, in ovarian cancer, high MKP1 expression levels correlate with a clear reduction in tumour recurrence-free survival [2]. In addition, it is also reported that MKP1 levels are highly upregulated after cisplatin treatment in ovarian cancer, and MKP1 knockdown promotes cisplatin mediated apoptosis in the same pathology [3]. Indeed, a similar role has been proposed in lung or breast cancer. Therefore, MKP1 was considered to confer oncogenic and chemo-resistance properties, suggesting that down-modulation could be a good therapeutic approach.

However, recent evidences indicate that MKP1 is a potential inducer of chemo-sensitivity in a very special context such as the presence of *E1a* gene. E1A is the first transcript of the early region of the adenovirus which is a key part of the infective cycle. It is responsible for regulating the transcription of the later adenoviral genes, promoting the entrance in S phase and the suppression of the defensive mechanisms of the host cell. E1A expression is also responsible for apoptosis induction in different experimental models, being able to collaborate in chemo and radiotherapy treatments. Recently, we observed that E1A was able to increase cisplatin sensitivity in some resistant NSCLC cell lines (as H1299) through MKP1 transcriptional upregulation mediated by E1A expression [4]. Indeed, MKP1 knockdown restores chemo resistance in E1A expressing H1299 cell lines, confirming how the axis E1A -> MKP1 -> p38MAPK promotes sensitivity, blocking the characteristic autophagic response associated to resistant models [5], and promoting an increase in the apoptotic onset. Indeed, inhibition of p38 has been also recently proposed as a mechanism of chemosensitivity in different *in vitro* and *in vivo* models [6]. Therefore, all these observations should be considered in a future therapy based in MKP1 inhibition. In this regard the obvious question is what will happen in a tumour in which the transformation is based in an E1A-like mechanism. It is clear that in this case MKP1 inhibition will promote resistance to conventional chemotherapy, as is the case of cisplatin. So, the next question is to know if an E1A-like mechanism is implicated in tumorigenesis. In this regard, we demonstrated several years ago how E1A was able to bypass Oncogene-Induced Senescence (OIS), as the induced by v-H-Ras, increasing MKP1 transcriptional levels, enabling in this way the nuclear inactivation of ERK1/2 [7]. Therefore, as the scape from OIS is a key event in cellular transformation, it seems to be highly probable that an E1A-like mechanism could be implicated.

Therefore, while MKP1 is a villain (oncogenic) in E1A mediated transformation, it could be also the good Samaritan able to promote chemosensitivity in resistant tumours. However, the important question goes beyond the experimental model based in E1A: should we use the inhibition of MKP1 in cancer therapy? We do strongly believe that MKP1 is a novel biomarker and a possible therapeutic target, but this observation could not be applied indiscriminately to any tumour. The presence of specific alterations could be implicated in the loss of the therapeutic benefits associated to MKP1 inhibition, as it may be the presence of a physiologic context similar to the one observed in the presence of E1A. In fact, it is noteworthy that alteration in key proteins present in many tumours, like p400, p300 or pRb, are also targeted by E1A to exert its transforming properties. In sum, MKP1 have two (or even more) faces, but we should manage to use the one closer to Dr. Jekyll and get rid of Mr. Hyde to fully explore its therapeutic potential.
